# Cell-Free Systems Enable the Production of AB_5_ Toxins for Diagnostic Applications

**DOI:** 10.3390/toxins14040233

**Published:** 2022-03-23

**Authors:** Franziska Ramm, Lena Jack, Danny Kaser, Jeffrey L. Schloßhauer, Anne Zemella, Stefan Kubick

**Affiliations:** 1 Fraunhofer Institute for Cell Therapy and Immunology (IZI), Branch Bioanalytics and Bioprocesses (IZI-BB), Am Mühlenberg 13, 14476 Potsdam, Germany; franziska.ramm@izi-bb.fraunhofer.de (F.R.); lena.jack@izi-bb.fraunhofer.de (L.J.); danny.kaser@izi-bb.fraunhofer.de (D.K.); jeffrey.schlosshauer@izi-bb.fraunhofer.de (J.L.S.); anne.zemella@izi-bb.fraunhofer.de (A.Z.); 2 Institute of Chemistry and Biochemistry—Biochemistry, Freie Universität Berlin, Takustr. 6, 14195 Berlin, Germany; 3 Faculty of Health Sciences, Joint Faculty of the Brandenburg University of Technology Cottbus–Senftenberg, Brandenburg Medical School Theodor Fontane and the University of Potsdam, 14476 Potsdam, Germany

**Keywords:** cholera toxin, heat-labile enterotoxin, AB_5_ toxins, eukaryotic cell-free systems, orthogonal systems

## Abstract

Cell-free protein synthesis (CFPS) represents a versatile key technology for the production of toxic proteins. As a cell lysate, rather than viable cells, is used, the toxic effects on the host organism can be circumvented. The open nature of cell-free systems allows for the addition of supplements affecting protein concentration and folding. Here, we present the cell-free synthesis and functional characterization of two AB_5_ toxins, namely the cholera toxin (Ctx) and the heat-labile enterotoxin (LT), using two eukaryotic cell-free systems based on Chinese hamster ovary (CHO) and *Spodoptera frugiperda (Sf*21) cells. Through an iterative optimization procedure, the synthesis of the individual AB_5_ toxins was established, and the formation of multimeric structures could be shown by autoradiography. A functional analysis was performed using cell-based assays, thereby demonstrating that the LT complex induced the characteristic cell elongation of target cells after 24 h. The LT complex induced cell death at higher concentrations, starting at an initial concentration of 5 nM. The initial toxic effects of the Ctx multimer could already be detected at 4 nM. The detection and characterization of such AB_5_ toxins is of utmost importance, and the monitoring of intracellular trafficking facilitates the further identification of the mechanism of action of these toxins. We showed that the B-subunit of LT (LTB) could be fluorescently labeled using an LTB-Strep fusion protein, which is a proof-of-concept for future Trojan horse applications. Further, we performed a mutational analysis of the CtxA subunit as its template was modified, and an amber stop codon was inserted into CtxA’s active site. Subsequently, a non-canonical amino acid was site-specifically incorporated using bio-orthogonal systems. Finally, a fluorescently labeled CtxA protein was produced using copper-catalyzed click reactions as well as a Staudinger ligation. As expected, the modified Ctx multimer no longer induced toxic effects. In our study, we showed that CFPS could be used to study the active centers of toxins by inserting mutations. Additionally, this methodology can be applied for the design of Trojan horses and targeted toxins, as well as enabling the intracellular trafficking of toxins as a prerequisite for the analysis of the toxin’s mechanism of action.

## 1. Introduction

Pathogenic bacteria continuously produce bacterial toxins, which exert various toxic effects in human beings. Some of these toxins are complex multi-component structures such as the class of AB_5_ toxins. These toxins are characterized by their structural composition, and as the name implies, such toxins consist of an A and B subunit. The A subunit, which is the catalytic subunit, and thus induces the toxic effect, can be split into two parts. The A1 subunit and the A2 subunit can be separated by a furin cleavage site, but the fragments subsequently remain linked via a disulfide bridge [1]. The A1 fragment contains the catalytic domain whilst the A2 fragment acts as an anchor to the B subunit. The B subunit forms a stable pentameric ring, which acts as the receptor-binding domain and attaches to the cell surface. When the A subunit is cleaved at the serine–protease cleavage site, the alpha helical A2 fragment anchors the A1 fragment to the core of the pentameric B ring. The A1 fragment is subsequently internalized into the cell [2,3,4].

Two of the best-characterized AB_5_ toxins are the cholera toxin (Ctx) and the heat-labile enterotoxin (LT). Infections with bacterial strains secreting these toxins into the intestinal lumen can cause mild-to-severe disease progressions. Watery stool or even severe diarrhea, dehydration and vomiting are typical symptoms of such an infection [4,5,6]. The B subunit of Ctx and LT generally target the monosialoganglioside GM1 receptor, which is expressed in jejunal epithelial cells [7]. Further studies have later shown that the B subunit is cross-reactive and that glycoproteins on the cell surface are responsible for the binding which leads to toxicity in the absence of GM1 [8,9]. After binding to the cell surface, Ctx as well as LT catalyze the ADP-ribosylation of the heterotrimeric G protein, specifically the Gs alpha subunit. This eventually leads to the continuous activation of adenylate cyclase and production of cyclic adenosine monophosphate (cAMP) [4,10]. Thus, the infected intestinal cell secretes chloride and water, leading to typical symptoms of diarrhea. In vitro assays confirmed the activation of the adenylate cyclase and showed that Chinese hamster ovary (CHO) target cells were elongated after intoxication with Ctx and LT, suggesting morphological changes attributed to the increased cAMP level [10,11].

Toxins, especially multi-component toxins, are “difficult-to-express” proteins, but studying and understanding the mechanism of action is mandatory to counteract toxic effects. An alternative to conventional in vivo expression systems is cell-free protein synthesis (CFPS). As a cell lysate is used, the synthesis of a toxin typically does not harm the expression system itself [12,13]. This is important when using eukaryotic systems for synthesis, as many bacterial toxins target eukaryotic cells and their signaling systems [4,14]. Further, using a eukaryotic cell-free system allows for the direct application of the protein of interest for functionality assessments avoiding prior purification steps, as the eukaryotic cell-free system enables the endotoxin-free production of the protein of interest [15]. The open system of CFPS enables the addition of factors affecting the functionality of the individual protein. Labeled amino acids can be added to the reaction mixture for quantitative and qualitative analysis. As the modification of toxins is of significance for studying the toxin’s characteristics, orthogonal translation systems can be applied. Such a system consists of an orthogonal synthetase, tRNA and a non-canonical amino acid (ncAA). The presence of an amber stop codon in the protein-encoding template and the subsequent implementation of the orthogonal systems allow for the fast and efficient modification of proteins via the incorporation of the individual ncAA which can further be labeled with fluorescent dyes [16,17]. Hence, cell-free protein synthesis is a qualified option to synthesize and characterize a wide variety of toxic proteins such as pore-forming and apoptosis inducing proteins [18,19,20]. The synthesis of AB_5_ toxins has generally been performed in prokaryotic cell-based systems such as via overexpression in *Escherichia coli* (*E. coli*) [1,10,21]. In this study, eukaryotic cell-free systems and orthogonal labeling systems were used to produce, modify and characterize the two AB_5_ toxins Ctx and LT. Two well established cell-free systems, namely the mammalian CHO-derived and the insect-*Sf*21-derived lysates, were used. It was further shown that CFPS could be used to develop a Trojan horse application as the LTB subunit was fused to Streptavidin (Strep) in silico, and after the synthesis of the fusion protein it was labeled with a biotin-conjugated fluorescent dye. Finally, the CtxA subunit was modified with an amber stop codon and ncAAs were incorporated using orthogonal systems. Subsequently, the subunit was fluorescently labeled using copper catalyzed click chemistry and a Staudinger ligation. Such mutational analysis studies and the combination with amber suppression and fluorescent labeling qualifies CFPS for the development of targeted toxins and for studying the intracellular trafficking of toxins, thereby identifying their mechanism of action. This is the first study using eukaryotic cell-free systems for studying and modifying Ctx and LT, therefore facilitating CFPS as a platform technology for analyzing the pathways of toxins such as AB_5_ toxins. 

## 2. Results

### 2.1. Cell-Free Synthesis of Ctx and LT

**Figure 1:**
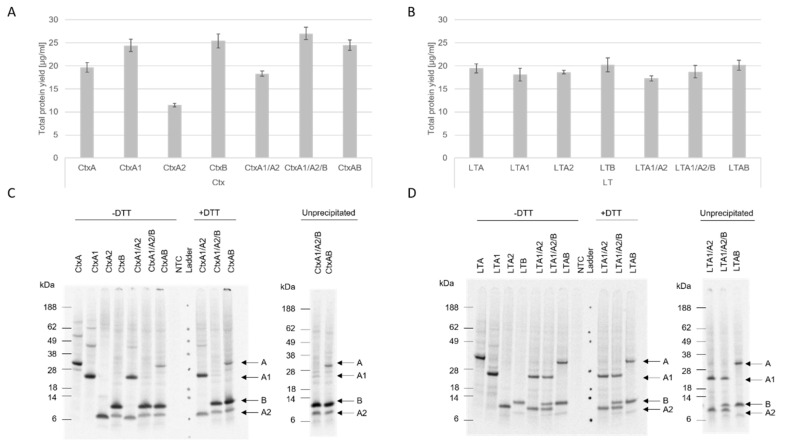
Cell-free synthesis of Ctx and LT. Single subunits and co-expressed subunits of Ctx (**A**) and LT (**B**) were synthesized in a CHO lysate. Total protein yields of *de novo* synthesized protein were analyzed by liquid scintillation for the translation mixture (TM). Standard deviations were calculated from triplicate analysis. Autoradiographs depicting ^14^C-labeled protein bands after a synthesis in CHO lysate for Ctx (**C**) and LT (**D**). A no-template control (NTC) was used as a negative control.

Until today, Ctx and LT were mainly synthesized in cell-based prokaryotic systems. As discussed, bacterial toxins such as AB_5_ toxins target eukaryotic cells and their signaling pathways, often impeding cell-based eukaryotic synthesis. Here, the cell-free syntheses of Ctx and LT subunits as well as their co-expression in two different cell-free systems were established. These eukaryotic cell-free systems were chosen, as eukaryotic systems in general are more favorable concerning post-translational modifications (PTMs) [13]. The CFPS of Ctx and LT was established in the mammalian CHO lysate and the insect-based lysate *Sf*21 in order to identify the most suitable lysate. The individual subunits as well as the co-expressions of AB, A1A2 and A1A2B were assessed. The CHO-based system resulted in slightly higher protein yields in comparison to the *Sf*21-based system. Considering the Ctx constructs synthesized in the CHO system, the A2 subunit resulted in the lowest overall protein yield at 11.5 µg/mL (Figure 1A). In the *Sf*21 lysate, CtxA2 and the co-expressed CtxA fragments resulted in the lowest total protein yields with 7.9 and 10.8 µg/mL, respectively (Figure S1A). The syntheses of LT constructs in a CHO system showed stable protein yields for all individual subunits and the co-expressions resulted in protein yields between 15 and 20 µg/mL (Figure 1B). In the *Sf*21 system, LTA2 was synthesized with the lowest overall yields of 9.5 µg/mL. All other syntheses of LT subunits as well as co-expressions resulted in stable yields between 10 and 15 µg/mL (Figure S1B).

Subsequently, the holotoxin formation (complete AB_5_ construct) was analyzed by autoradiography. Therefore, the proteins were applied on SDS-PAGEs in different manners. Acetone-precipitated samples were solved in sample buffer with and without the presence of the reducing agent dithiothreitol (DTT) and compared to unprecipitated samples. All subunits could be detected by autoradiography. After a synthesis based on CHO lysate, high-molecular-weight bands for CtxA and CtxA1, in addition to the protein bands of CtxA and CtxA1, could be detected. Less-intense high-molecular-weight bands were visible for LTA and LTA1 (Figure 1C,D). CtxB showed multimerization after a CHO and *Sf*21 synthesis, indicating the formation of a pentameric ring formation (CHO Figure 1C, *Sf*21 Figure S1C). Strikingly, when co-expressing CtxA1, CtxA2 and CtxB, only a minor A1 protein band could be detected in all samples. This could suggest that, in a co-expression of all three subunits, the syntheses of the A2 and B subunits were favored. The presence of the complete A subunit is essential for the toxin’s functional activity; therefore the co-expression of the complete A subunit with the B subunit was preferentially used in the functional assessments performed later on. Complete AB_5_ multimer formation was not detectable in the autoradiograph. The autoradiograph of the unprecipitated sample of the co-expressed CtxA1 and A2 subunit in an *Sf*21 system did not only detect the individual protein bands for the A1 and A2 fragment, but also showed a clear protein at the molecular weight of the full length A subunit (Figure S1C). A strong signal peptide cleavage was seen for LT fragments in the *Sf*21 system (Figure S1D).

Overall, these data showed that eukaryotic cell-free systems were able to successfully synthesize individual subunits and co-express the subunits of Ctx and LT. Multimerization of the single subunits indicated that the assembly of the AB_5_ toxin is possible in cell-free systems, although no holotoxin formation could be detected in the autoradiograph.

In the next step, protein modifications were performed on the individual subunits. In prior studies, LTB was described as more resistant to external factors [22]. Therefore, we chose the B subunit of LT to be modified rather than CtxB to investigate fusion proteins of the B subunit. As the A subunit from Ctx was shown to be more potent than LTA [23,24], we aimed to insert two mutations in the active center of CtxA to assess whether CFPS is suitable to study the toxic effects of mutants. 

### 2.2. Modification of LTB

**Figure 2:**
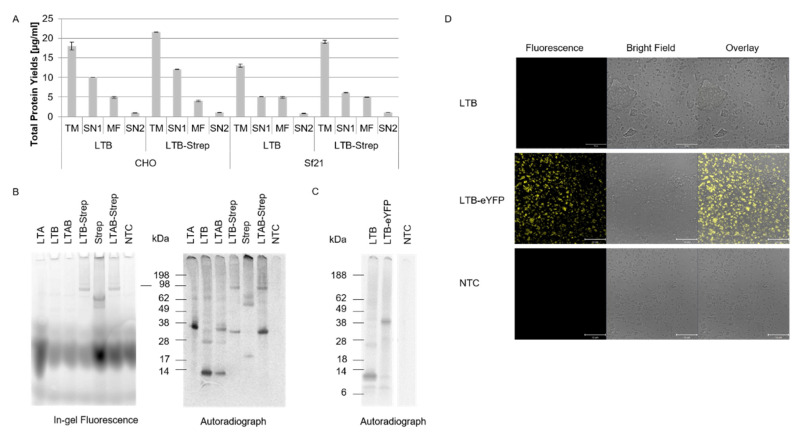
Modification of the LTB subunit. (**A**) Determination of total protein yields in each fraction for LTB and LTB-strep after a synthesis in a CHO and *Sf*21 lysate. Total protein yields of *de novo* synthesized protein were analyzed by liquid scintillation for translation mixture (TM), the first supernatant (SN1), the microsomal fraction (MF) and the translocated soluble protein in the second supernatant (SN2). Standard deviations were calculated from triplicate analysis. (**B**) LTA, LTB, LTAB, LTB-Strep, Strep, LTAB-Strep and the no template control (NTC) were synthesized in the *Sf*21 system and fluorescently labeled with a Biotin-Atto 488 dye. In-gel fluorescence showing the Atto-488-labeled protein bands and autoradiograph depicting the ^14^C-labeled bands. (**C**) Autoradiograph depicting the ^14^C-labeled protein bands after the 3^rd^ round of repetitive synthesis in an *Sf*21 lysate for LBT, LTB-eYFP and the NTC. (**D**) CLSM images for LTB, LTB-eYFP and the NTC after three rounds of synthesis in an *Sf*21 lysate.

The LTB subunit was modified in two different ways. A fusion construct of the LTB subunit and a streptavidin was designed, where Strep was fused to the C-terminus of LTB. AB_5_ toxins form stable multimers that are linked by disulfide bridges and non-covalent bindings [4]. In eukaryotic cell-free systems, PTMs such as disulfide bridges are generally performed in the vesicles from the endoplasmic reticulum (ER) [13]. Hence, the co-translational translocation for these proteins was important, and thus all constructs harbored a melittin (Mel) signal peptide. In order to compare the synthesis efficiency of the LTB subunit and the LTB-Strep fusion construct, both constructs were synthesized in the two eukaryotic lysates and analyzed for their translocation efficiency into ER-based vesicles. Therefore, after cell-free synthesis, the translation mixture (TM) was fractionated into the soluble supernatant (SN1) and the translocated soluble components (SN2) within the microsomal vesicles. The total protein yield was determined, and the data indicated that both eukaryotic systems similarly translocated both proteins into the vesicles (Figure 2A). The total protein yield in the TM was generally higher in CHO lysate, but the translocated soluble protein was higher in the *Sf*21 syntheses. In the CHO system, 5.3% of the total protein yield of LTB and 4.9% of LTB-Strep were translocated and could be released from the vesicles. In the insect-based system, 6.1% and 5.9% could be retrieved for LTB and LTB-Strep, respectively. The AB_5_ holotoxin complexes are thought to rely on disulfide bridging [1], thus the translocation efficiency of the cell-free system into the ER-based vesicles is mandatory for a more stable complex formation. Based on the results of LTB and LTB-Strep in CHO and *Sf*21 lysate, the insect-based *Sf*21 system was chosen for further experiments with LT constructs.

After the synthesis of the LTB fusion construct, the biotin-conjugated fluorophore Atto 488–Biotin was coupled to LTB-Strep. All single subunits and co-expressed LTAB, as well as co-expressed LTAB-Strep and a Strep molecule alone were labeled with the fluorophore. The 16.5 kDa Strep molecule alone displayed a higher molecular weight than the LTB monomer. The in-gel fluorescence showed fluorescent bands between 17 and 38 kDa in all lanes indicating the running front of the biotin conjugated fluorophore. Nonetheless, defined, high-molecular-weight bands around 98 kDa could be detected for LTB-Strep, Strep and LTAB-Strep, which might suggest a multimerization of the Strep molecules. Autoradiography confirmed these protein bands, and thus indicated a protein-specific labeling with the biotin conjugated fluorophore. The autoradiograph additionally showed lower-molecular-weight bands that were not detectable in the in-gel fluorescence (Figure 2B).

In order to visualize the co-translational translocation of the protein, a further construct was developed by a two-step polymerase chain reaction (PCR). The gene construct for LTB was fused to the gene sequence of the enhanced yellow fluorescent protein (eYFP). With the aim of enhancing the translocation efficiency, a repetitive synthesis of LTB, LTB-eYFP, and the no-template control (NTC) was performed. After three rounds of syntheses, the autoradiograph showed specific protein bands for the respective proteins but not the NTC (Figure 2C). Using confocal laser scanning microscopy (CLSM), the microsomal vesicles were visualized. No fluorescence could be detected for LTB alone and the NTC. The fluorescence of the LTB-eYFP fusion protein, in comparison to the NTC and LTB alone, clearly showed a strong difference in the fluorescence of the microsomal vesicles (Figure 2D).

**Figure 3:**
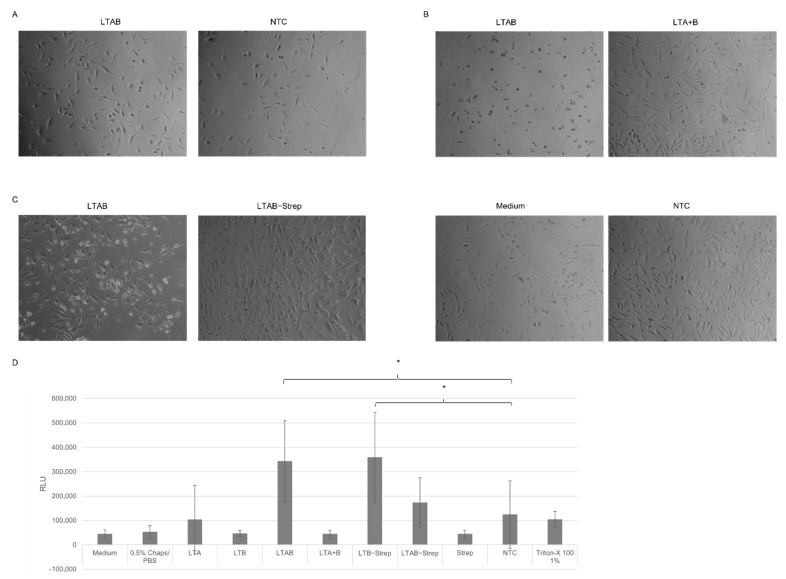
Morphological analysis of CHO-K1 cells after LT addition. Co-expressed LTAB and pre-synthesized LTA and B, which were mixed afterwards to form the multimer (LTA + B), were synthesized in *Sf*21 lysate and supplemented to CHO-K1 cells (4,000 cells/well) in a 96-well plate. Untreated cells and cells supplemented with a no-template control (NTC) in a volume equivalent to the highest toxin concentration served as controls. Phase contrast photographs were taken. (**A**) LTAB and the volume-equivalent NTC were supplemented at 3 nM and documented after 24 h. (**B**) LTAB, LTA + B and the volume-equivalent NTC were supplemented at 6 nM and documented after 48 h. (**C**) Co-expressed LTAB and LTAB-Strep were synthesized in *Sf*21 lysate and supplemented to CHO-K1 cells (25,000 cells/well) at 5 nM in a 24-well plate. Phase contrast photographs were taken after 48 h of incubation. (**D**) Analysis of cytotoxic behavior of LT and its subunits on CHO-K1 cells after 48 h incubation in a 96-well plate detected by relative light units (RLU). Cells supplemented with medium; 0.5% Chaps/PBS or a NTC in an equivalent volume served as negative controls and Triton-X 100 at 1% served as a positive control. Standard deviation derived from three assays with triplicate analysis (*n* = 9). Statistical significance by ANOVA according to Bonferroni, as indicated by *.

These data showed that the *Sf*21 lysate was well-suited for the analysis of LTB fusion proteins and the functional analysis of the LT complex and its individual subunits; the modified LTB-Strep protein was further investigated in *Sf*21 lysate. Morphological changes in CHO-K1 cells were studied. Prior studies depicted that AB_5_ toxins induced morphological changes after the incubation with target cells even after 4 to 24 h [10,11]. Hence, a concentration gradient of the unmodified LT complex and its individual subunits in a cell-based assay was assessed after 24 and 48 h. These data showed that, at lower concentrations (2 and 3 nM), the co-expressed LTAB started to induce morphological changes after 24 h in contrast to NTC-treated cells. At 3 nM, elongated CHO-K1 target cells could be detected (Figure 3A). Higher concentrations of 5 and 6 nM showed that cells did not efficiently adhere in the presence of LTAB, suggesting toxic effects at higher concentrations (Figure S2). The effects of the toxin at the different concentrations were additionally assessed after 48 h. The co-expressed LTAB at a concentration of 6 nM presented the strongest effects, inducing morphological changes and detachment of cells, which suggested cell death. When LTA and B were individually synthesized and mixed together after the synthesis, these effects were not detected, which could demonstrate the necessity of disulfide bridges for complex formations. Untreated and NTC-treated cells did not show any effects (Figure 3B). Firstly, morphological changes could be observed at a concentration of 5 nM for cells treated with co-expressed LTAB but not at 2 and 3 nM (Figure S3). Single subunits did not show morphological changes in cells at the highest concentrations (Figure S3).

Subsequently, we investigated whether the LTB-Strep construct was also able to form a functional complex when co-expressed with LTA. Hence, the co-expressed subunits of the wild-type (WT) and Strep-modified construct, as well as the individual subunits, were supplemented to CHO-K1 cells. This time, a concentration of 5 nM was applied as the prior experiments showed that 5 nM induced major morphological changes. The data reflected that a concentration of 5 nM LTAB multimer induced initial morphological effects after 48 h. The LTAB-Strep complex did not show such effects (Figure 3C). Single subunits as well as Strep alone did not induce characteristic morphological changes (Figure S4). To quantify the activity of the individual subunits and the holotoxins, a cytotoxicity assay was performed. The toxins were supplemented at a concentration of 6 nM to assess the toxic nature. In this assay, high relative light units (RLU) indicated toxicity. Interestingly, high values were not only detected for LTAB but also for LTB-Strep and the NTC. In comparison to the NTC lysate control, LTAB and LTB-Strep induced statistically significant changes (Figure 3D). No other sample showed significant changes in comparison to the NTC. No significant difference between LTAB and LTB-Strep could be detected. As shown in Figure S5 no effects of LTB-Strep on the cells could be detected. Hence, these higher RLU might correspond to background noise from the lysate. These data suggest that the modification of the B subunit leads to a reduced functionality.

### 2.3. Incorporation of ncAAs into CtxA

**Figure 4:**
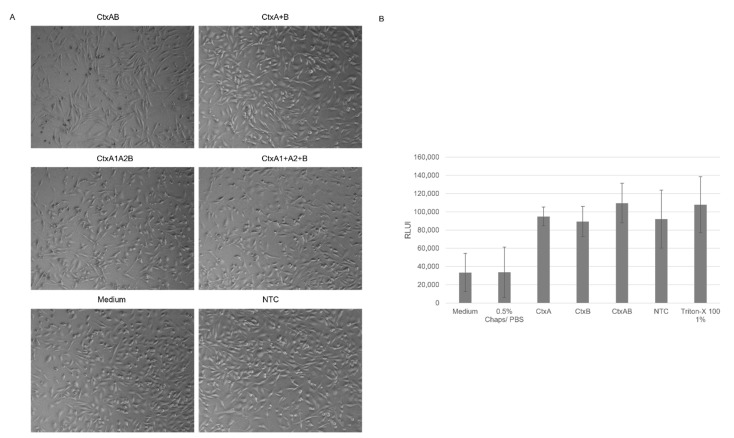
Morphological analysis of CHO-K1 cells supplemented with Ctx toxin. Ctx subunits, co-expressed CtxAB and CtxA1A2B complexes were synthesized in CHO lysate. Co-expressed CtxAB and CtxA1A2B and mixed complexes (CtxA + B and CtxA1 + A2 + B) were investigated. (**A**) Toxins were supplemented to CHO-K1 cells (25,000 cells/well) in a 24-well plate. Untreated cells and cells supplemented with NTC in an equivalent volume served as controls. Phase contrast photographs were taken after 48 h of incubation. (**B**) Analysis of cytotoxic behavior of Ctx and its subunits on CHO-K1 cells after 48 h incubation in a 96-well plate detected by relative light units (RLU). Cells supplemented with medium, 0.5% Chaps/PBS or a NTC in an equivalent volume served as negative controls and Triton-X 100 at 1% served as a positive control. Standard deviation derived from three assays with triplicate analysis (*n* = 9).

The functional activity of Ctx was assessed after a synthesis in CHO and *Sf*21 lysate. CtxAB and its individual subunits were supplemented to CHO-K1 cells, and the induced changes were analyzed after 48 h. In addition to the co-expressed AB multicomponent protein and the subunits alone, A1A2B co-expression was investigated. As shown in Figure 1, lower total protein yields could be detected for certain subunits, such as CtxA2. In order to supplement all toxin fragments at the same concentration, all compounds were diluted to a final concentration of 4 nM. After a synthesis in a CHO lysate, the AB and the A1A2B co-expressed subunits induced initial morphological changes after 48 h of incubation. When the subunits were individually synthesized and mixed together afterwards, no effects on the target cells could be detected 48 h after supplementation (Figure 4). Single subunits did not induce any morphological changes (Figure S6). After a synthesis in *Sf*21 lysate, the supplementation of co-expressed CtxAB also resulted in morphological changes in CHO-K1 cells (Figure S7). These data indicate the suitability of both lysates for the analysis of Ctx. A quantification of cytotoxic behavior was assessed by using the CellTox green cytotoxicity assay for Ctx synthesized in CHO lysate. A concentration of 4 nM was used again. High RLU were detected for the individual samples. The values for CtxAB were higher than for CtxA or CtxB, but high background noise could be detected again. No morphological changes could be detected for CtxA, CtxB or the NTC, but changes could also be seen for CtxAB (Figure S8). The statistical analysis showed that no sample was significantly different from the NTC lysate background indicating an interaction between the assay reagent with the CHO lysate. Hence, background signals from the lysate could be detected.

**Figure 5:**
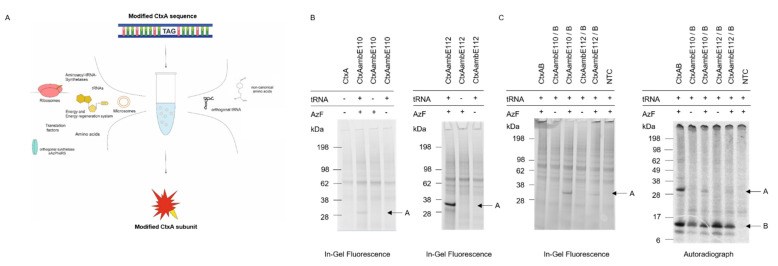
Synthesis of CtxAamb mutants and labeling using orthogonal systems. (**A**) Schematic overview of the orthogonal translation. (**B**) In-gel fluorescence of CtxAamb mutants labeled with DyLight-632 phosphine after orthogonal translation in an optimized CHO system. (**C**) In-gel fluorescence and autoradiography of co-expressed multimer after the incorporation of AzF and subsequent labeling with DyLight-632 phosphine.

As a final modification, the A subunit of the cholera toxin was modified, and two mutants were designed, harboring an amber stop codon instead of a glutamic acid codon at either position 110 or 112 in the enzymatic center of the catalytic subunit. The constructs were termed CtxAambE110 and CtxAambE112. The amber stop codon allowed for the site-specific incorporation of ncAAs using orthogonal systems (Figure 5A). The proof of concept for the incorporation of ncAAs into the CtxA subunit by using a PCR template was performed in both eukaryotic lysates (CHO and *Sf*21). The initial incorporation efficiency was not sufficient, and thus the reaction parameters were optimized (data not shown). As a cell-free system is an open system, PCR templates encoding both CtxA and CtxB as well as all the components in the orthogonal system had to be added to the reaction, which led to a reduced amount of template within the reaction mixture. A novel CHO lysate that integrated the aminoacyl-tRNA synthetase eAzPheRS was tested and showed the most promising results. As the aminoacyl-tRNA synthetase was integrated in the lysate, no further supplementation of this enzyme was necessary. This resulted in higher amounts of PCR templates that could be supplemented to the reaction mixtures. Using this set up, fluorescently labeled protein bands for the CtxA mutants could be detected when orthogonal tRNA and an ncAA was added, and this lysate was used for all further analyses. As expected, no fluorescently labeled band could be detected when one component was missing in the control reactions. This was shown for both mutated constructs (CtxAambE110 and CtxAambE112) when the ncAA p-azido-L-phenylalanine (AzF) was incorporated, and the proteins were subsequently labeled with DyLight-632 phosphine (Figure 5B). Constructs that incorporated p-propargyloxyphenylalanine (pPa) were labeled in a copper-catalyzed click reaction. Similar results were observed for the copper-catalyzed click reaction of modified CtxA subunits that incorporated pPa, and were labeled with sulfo-Cy5-azide (Figure S9). If a label, a specific targeting or toxic moiety were to be clicked to the modified CtxA subunit and applied to cell-based assays, copper might harm the cells. Therefore, the linkage between an azide of AzF and a phosphine group, called Staudinger ligation, provides an alternative copper-free method. Consequently, the incorporation efficiency of AzF was further investigated. The in-gel fluorescence of both modified CtxA subunits when co-expressed with CtxB in the presence of AzF showed intensely labeled protein bands. No fluorescence signal was detected when AzF was missing from the synthesis and when wild-type (WT) CtxA and the NTC were labeled. In the autoradiograph, all lanes depicted strong protein bands for the CtxB subunit. After the CtxA WT subunit was co-expressed with CtxB (CtxAB) a strong protein band could also be detected for CtxA. A less intense protein band for the CtxAambE110 construct was visualized after a synthesis in the presence of AzF. The CtxAambE112 construct only presented a minor full-length band in the presence of AzF. As expected, no full-length CtxA protein bands were detected when AzF was absent in the reaction as the ncAA could not be incorporated. The termination products of the CtxA proteins could not be detected as they are about the same size as the CtxB monomer (Figure 5C). This indicated that the incorporation efficiency using AzF and the modified CHO system were better for the CtxAambE110 construct, but the translation efficiency was reduced compared to the WT CtxA fragment, thereby leading to less intense protein bands in the autoradiograph.

**Figure 6:**
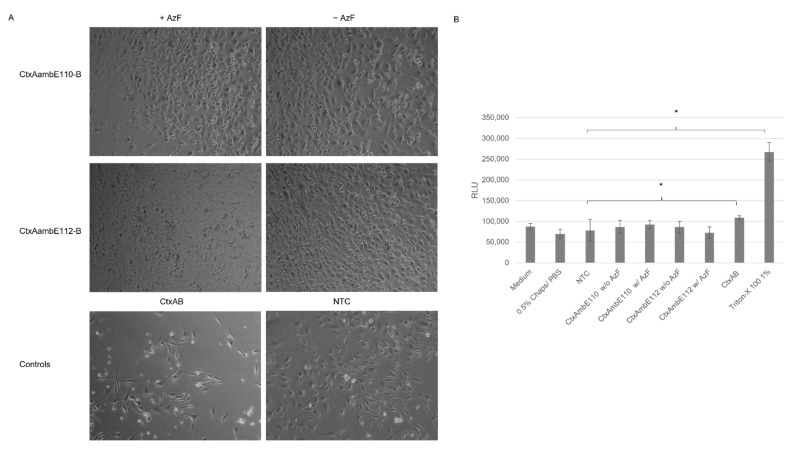
Analysis of CHO-K1 cells supplemented with Ctx toxin with a modified A subunit. Ctx subunits were co-expressed in a modified CHO lysate. (**A**) Toxins were supplemented to CHO-K1 cells (25,000 cells/well) in a 24-well plate at a concentration of 4 nM. Phase contrast photographs were taken after 48 h of incubation. (**B**) Analysis of cytotoxic behavior of modified CtxA subunits on CHO-K1 cells after 48 h incubation in a 96-well plate detected by relative light units (RLU). Cells supplemented with medium, 0.5% Chaps/PBS or a NTC in an equivalent volume served as negative controls and Triton-X 100 at 1% served as a positive control. Standard deviation derived from three assays with triplicate analysis (*n* = 9). Statistical significance by ANOVA according to Bonferroni, as indicated by *.

Subsequently, the effect of the modified CtxA mutants on CHO-K1 cells was determined. The CtxB subunit was co-expressed with the CtxA WT or one of the mutants. The reactions were carried out with the modified CHO lysate in the presence of AzF and orthogonal tRNA for WT, mutants and NTC. The syntheses of the two mutants were additionally performed without the presence of AzF as a negative control. Untreated cells were at a maximum confluence after 48 h, while the NTC showed a reduced confluence. At 4 nM, CtxAB inflicted initial morphological changes of cells. Cells that were supplemented with a mutated A subunit were very confluent, both when AzF was added to the synthesis reaction and when AzF was not added to the mixture (Figure 6A). In order to demonstrate the toxic effects of the Ctx complexes a cytotoxicity assay was performed. High fluorescence values indicate cytotoxicity. These data indicated that the CtxAB multimer induced initial cytotoxic effects on the cells, while the modified CtxA multimers did not. In comparison to the NTC lysate control, CtxAB but not the modified holotoxins induced statistically significant changes. As indicated by * (Figure 6B). These data suggest that the incorporation of a ncAA in the enzymatic center of the toxin inhibited the functionality of the toxin.

Taken together, the data presented in this study demonstrate that CFPS is a versatile method to synthesize, modify, detect and investigate Ctx and LT and modified variants. The data acquired here show the versatility of CFPS for the design of targeted toxins and Trojan horses and its use for analyzing the mechanism of action of these toxins.

## 3. Discussion

A major group of toxins expressed by pathogenic bacteria is the group of AB_5_ toxins. This group of toxins is rather diverse and was therefore subcategorized into different families. After the detection of the novel AB_5_ toxin subtilase, a new family was announced in the early 2000s [25], showing that there is an increasing demand for stable and easy-to-handle production systems for the synthesis and characterization of active AB_5_ toxins. The two best-characterized and most-well-known representatives of this group are Ctx and LT. Our study aimed to synthesize, characterize and modify Ctx and LT in eukaryotic cell-free systems. In prior studies, Ctx and LT were isolated from prokaryotic cell-based systems [1,10,21]. Prokaryotic cell-free systems generally lack the ability to form PTMs such as disulfide bridges. The addition of supplements, such as a redox system, is necessary to functionally synthesize active proteins containing selected PTMs. As bacterial strains can further encode endotoxins, proteins expressed in prokaryotic systems must be purified for further analyses. A eukaryotic cell-free system circumvents these drawbacks [13].

Our data showed that both eukaryotic systems, CHO and *Sf*21, resulted in reproducible total protein yields and functional activity. Nonetheless, higher protein yields were detected in the CHO system. In prior work, we demonstrated that the CHO system can be used as a high-yield system [12], thus our data add to prior findings that different toxin subunits with diverse molecular weights can be efficiently synthesized in eukaryotic systems, especially the CHO system. Strikingly, the CtxA2 subunit resulted in the lowest protein yields in both CHO and *Sf*21, and the LTA2 subunit showed similarly low yields in the *Sf*21 system. These data indicate that the A2 subunit is difficult to express in comparison to the other subunits, which might be caused by its small size. Other studies generally expressed A2 chimeras with other toxin compounds and investigated the holotoxin formation [23,26], thus data on the synthesis of A2 itself are rather limited and future studies should investigate the single synthesis of A2 fragments.

The structure of AB_5_ toxins is characteristic for this class of toxins. The A subunit is cleaved into the A1 and A2 fragment which are subsequently linked together via a disulfide bridge [1]. Further, the A2 fragment non-covalently links the A subunit to the stable pentameric ring [4]. The data presented here indicate no holotoxin formation after an SDS-PAGE. Few multimerizations of the single subunits were detected. Jobling et al. showed a clear holotoxin formation of the AB_5_ multimer [27]. As these proteins were purified from *E. coli* strains, a higher total protein yield might have been loaded onto the gel. Prior studies have also shown that CtxB and LTB form two types of multimers with either a native or a non-native conformation. The non-native multimers appear to occur at acidic pH values and form different secondary conformations as compared to native multimers. While both types can bind to the receptor, the native subunit multimers are SDS-resistant, but SDS degrades the non-native multimers [22,28]. Additionally, Zrimi and colleagues identified that 35 µM of CtxB only showed a pentameric state, while a concentration of 8.6 µM led to monomeric states [22]. As shown in this study, slight multimeric structures in the CtxB subunit were detected, suggesting that a higher protein yield might be needed to visualize the pentameric state. In the case of LTB, no multimerizations were detectable in the autoradiograph. Either LTB only formed non-native multimers, which cleaved to the monomeric state in the SDS-PAGE, or the concentration of the formed multimers was not yet sufficient to be detected by autoradiography.

The cell-based assays showed that cell-free synthesized AB_5_ toxins induced characteristic morphological elongation of cells at lower concentrations after 24 h, which is in line with previous findings, where elongated cells were detected after 4 h and 24 h of incubation [10,11]. After 48 h, Ctx and LT induced detached cells and morphological changes suggested non-viable cells. A cytotoxicity assay was performed in order to quantify these changes. Strikingly, LTB-Strep and the individual Ctx subunits demonstrated high background values. These background signals could derive from unspecific interactions with the lysate as well as mRNA present in the sample. Different background values for the individual lysates could be detected suggesting the possibility of purification steps for this particular cytotoxicity assay in order to specifically compare the individual experiments. Nonetheless, the cytotoxicity data and cell micrographs indicate that functional AB_5_ complexes can be synthesized using cell-free protein synthesis. Complex toxin structures were active after cell-free co-expression of the subunits but not when mixing the subunits after their individual syntheses. This could indicate the importance of the formation of multimeric complexes by disulfide bridges and stable bonds for functionally active proteins. Prior work has shown that CHO and *Sf*21 lysates can be used to synthesize proteins that include disulfide bridges [29,30]. Thus, the formation of disulfide bonds in the AB_5_ complex of cell-free synthesized Ctx and LT could have led to functional proteins when co-expressing the subunits. When the individual subunits were mixed together following their individual syntheses, the complex formation seemed to be inefficient and did not lead to functional complexes. In addition to this, prior studies have also shown that free CtxA and CtxB subunits do not assemble to complete holotoxins [31]. If we assume this is also true for LT, our data add new information to the assembly of LT and clearly align with the findings for Ctx. Ctx is supposed to be the more potent toxin when comparing Ctx and LT, as Ctx forms more stable holotoxins [23,24,32]. Nonetheless, the pentameric B ring of LT was shown to be more resistant to external factors [22]. Despite these findings, no differences in the activities of WT holotoxins could be observed from the cell-free, synthesized holotoxins generated in this study.

The modified toxins used in this study did not show the same activity as WT proteins. When LTB was fused to Strep, the activity was decreased, which suggests that the holotoxin formation was not as stable as with WT LTB. As LTA is already less potent than CtxA [23,24,32], the modified LTB subunit might have led to a further decrease in activity in comparison to the WT construct. The fusion of the Strep molecule to the C-terminus of the LTB might have led to a reduced multimerization of the B subunit. The mutated CtxA subunits showed no effect on CHO-K1 cells. As both mutations were situated within the active center of the fragment, this outcome was expected. Prior studies have already investigated this active core and mutated the same or similar positions. Position E112 has been widely investigated as a non-toxic mutant for adjuvant applications and a mutation at E112 was shown to lead to a reduced toxicity [33,34]. Aligning with these previous studies, our findings demonstrated that a single mutation within the active center disrupts the toxins functionality. Silencing a toxin with a point mutation in cell-free systems will be beneficial for future studies. This will allow for the defined analysis of the toxins pathway within the targeted cell. 

In order to inhibit the toxic effect of a pathogen, the mechanism of action of the underlying toxin has to be fully understood. Tracking the toxin within the cell enables the clarification of the pathogenesis of diverse novel toxins. Few studies attempted to fluorescently label toxins. The labeling of proteins by generating a fusion protein with a fluorescent protein such as YFP leads to enlarged proteins, which might affect the activity of the protein. As shown in our study, the 12 kDa LTB is significantly smaller than eYFP. The LTB-eYFP fusion protein could still be translocated into the microsomal vesicles, indicating that larger fusion proteins might be a powerful analytical tool. Another study could even track the C-terminal fragment of tetanus toxin within the cell after it was fused to the green fluorescent protein [35]. A further idea to label a toxin fragment is to fuse the protein to streptavidin and, subsequently, couple a biotin-conjugated fluorophore to the protein. Such couplings of streptavidin–biotin are very strong and do not require the addition of further chemicals. Studies have even shown that cell-staining and antigen-labeling is possible within such systems [36]. All possible fluorophores and payloads that are linked to biotin can be “clicked” to the Strep modified construct, allowing for the diverse modifications of a single protein. The modified LTB-Strep construct in our study could be labeled with a biotin-conjugated fluorophore and was further detected by in-gel fluorescence. Thus, in future experiments, the fluorescent dye can easily be exchanged by a toxic moiety coupled to the B subunit. Under these conditions, the B subunit may further act as a Trojan horse, targeting the cells, and inducing a specific effect by the coupled moiety.

A major disadvantage of these systems is that the label has to be fused to the protein of interest either at the N- or at the C-terminus. This might especially be a drawback in multicomponent proteins such as AB_5_ toxins as the assembly of the complex might be hindered. Nonetheless, such fusion proteins were also tested for Ctx and LT, and it was shown that the labeled B subunits assembled and attacked the cells [37,38], suggesting that larger fusion proteins were possible. An alternative to such fusion proteins are site-specifically labeled proteins. In 2020, a study showed the incorporation of ncAAs into microcystins and the subsequent labeling of these constructs with copper-catalyzed click chemistry in living cells. Autofluorescence signals and unspecific labeling were detected but could be distinguished from specifically labeled peptides [39]. 

These data show that bio-orthogonal systems can be used for studying intracellular trafficking. The data presented in our study add to these findings, as a site-specific fluorescent label could be added to the CtxA subunit by using an amber stop codon and an orthogonal system. As indicated in Figure 5C, the full-length protein of the CtxA subunit harboring the incorporated ncAAs showed a reduced protein band in the autoradiograph as compared to the WT CtxA subunit. Nonetheless, the data acquired here show that small reaction volumes were sufficient for studying the toxicity of the different proteins. As CFPS is a scalable technology, small reaction volumes can be applied for parallel screenings, such as toxic mutants. The upscaling of cell-free reactions to mL or liter batches was shown in prior studies, thus facilitating CFPS for further down-stream processes [40,41].

Overall, the data presented in this study show that CFPS represents a versatile platform for the synthesis, characterization and modification of not only AB_5_ toxins but also proteinaceous toxins in general. Mutational studies can be performed in a high-throughput manner using cell-free systems as an amber stop codon can be inserted at any desired position within the gene of interest, thus enabling the screening of toxic domains. 

CFPS will facilitate the investigation of the mechanism of action of novel toxins by diverse labeling methods and subsequent intracellular trafficking in the future. The modification and conjugation of individual toxin subunits will also improve the development of targeted toxins and Trojan horses for therapeutic use. Overall, this study demonstrated that cell-free systems enable the production of toxins for diagnostic and therapeutic applications using the example of the AB_5_ toxins, Ctx and LT.

## 4. Materials and Methods

### 4.1. DNA Template Design

Gene sequences encoding the cholera toxin A subunit, the partial A1 sequence, the partial A2 sequence (UniProt accession: P01555) and the B subunit (UniProt accession: P01556), as well as gene sequences encoding the A and B subunit (UniProt accession: P06717 and P32890, respectively) of the heat-labile enterotoxin, were modified for cell-free protein synthesis. Therefore, the DNA templates were modified according to Brödel et al. [42] and the native signal sequences were replaced with the Melittin signal sequence. LTA was modified with a C-terminal double strep tag. These sequences were obtained by *de novo* gene synthesis (Biocat GmbH, Heidelberg, Germany), cloned into the pUC57-1.8K vector backbone and plasmids were directly used for cell-free synthesis. 

For further analyses, several additional templates were generated using an expression PCR (E-PCR) [43]. All constructs were amplified using an E-PCR with the HiFidelity polymerase and its corresponding kit (Qiagen GmbH, Hilden, Germany). Standard HiFi PCR protocol (f.c. 0.2 ng/µL DNA, 1x HiFi Buffer including dNTPs, 0.05 U/µL HiFi polymerase). After each individual PCR, the templates were analyzed by agarose gel electrophoresis.

### 4.2. LTA1 and LTA2 Templates

At first, LTA1 and LTA2 were additionally generated. For LTA1, the forward primer N0 (5′-ATGATATCTCGAGCGGCCGCTAGCTAATACGACTCACTATAGGGAGAC CACAACGGTTTCCCTCTAGAAATAATTTTGTTTAACTTTAAGAAGGAGATAAACAATG-3′) and the gene-specific reverse primer, X-LTA1-oe-C0-R (5′-CTTGGTTAGTTAGTTATTAGATTGTTCTTGATGAATT-3′), which included an overhang to the regulatory sequences, were required for CFPS. In a second step, the generated PCR template was amplified with N0 and reverse C0 (5′-ATGATATCACCGGTGAATTCGGATCCAAAAAACCCCTCAAGACCCGTTTAGAGGCCCCAAGGGGTACAGATCTTGGTTAGTTAGTTATTA-3′). For the LTA2 template, a gene-specific primer with an overhang to the regulatory sequences for CFPS X-NCM-oe-LTA2-F (5′-TACATTTCTTACATCTATGCGGACACAGGTGATACTTGTAAT-3′) and C0 were used in the first step. Next, the template was fused to the NCM-F primer that included the CrPV IRES site and the Mel signal peptide 

(5′-ATGATATCTCGAGCGGCCGCTAGCTAATACGACTCACTATAGGGAGACCACAACGGTTTCCCTCTAGAAATAATTTTGTTTAACTTTAAGAAGGAGATAAACAAAAGCAAAAATGTGATCTTGCTTGTAAATACAATTTTGAGAGGTTAATAAATTACAAGTAGTGCTATTTTTGTATTTAGGTTAGCTATTTAGCTTTACGTTCCAGGATGCCTAGTGGCAGCCCCACAATATCCAGGAAGCCCTCTCTGCGGTTTTTCAGATTAGGTAGTCGAAAAACCTAAGAAATTTACCTGCTAAATTCTTAGTCAACGTTGCCCTTGTTTTTATGGTCGTATACATTTCTTACATCTATGCGGAC-3′).

The templates from the first PCR step and the NCM-F primer were applied in an equal molar template ratio of 12.5 nM in a standard HiFi PCR protocol. 

### 4.3. Modification of Wild-Type Constructs

In the next step, the LTB gene was fused to the streptavidin (Strep) gene (UniProt accession: P22629) in silico. Strep was fused to the C-terminus of the LTB gene. Two mutated CtxA subunits were designed by exchanging the glutamic acid codon at position 110 or 112 (amino acid number counting without the signal peptide) to an amber stop codon (TAG). The modified LTB-Strep construct, as well as the mutated CtxA constructs (CtxAambE110 and CtxAambE112), were also obtained by *de novo* gene synthesis (Biocat GmbH) as a lyophilizate. The standard HiFi protocol was performed while applying the N0 and C0 primers.

A fusion construct of LTB with the fluorescent protein eYFP was generated to assess the co-translational translocation in the cell-free system. Therefore, the LTB template was modified by using the HiFi PCR scheme. In the first step, the N0 forward primer and the gene-specific reverse primer with an overhang to the eYFP X-LTB-oe-eYFP-R (5′-CTTGCTCACCTcTAGAcAGTTTTCCATACTGATTGCCGC-3′) were utilized. In a second step, the template was fused to an eYFP construct by mixing the two templates in a 1:1 molar ratio in a HiFi PCR and using N0 and C0 primers.

### 4.4. Strep Construct

In order to compare the synthesis of the modified LTB-Strep, not only to LTB but also to Strep itself, a 2-step E-PCR was performed. The Strep template was amplified from the LTB-Strep plasmid. Therefore, a standard HiFi PCR scheme, as described above, was applied using a gene-specific forward primer for Strep NCM-oe-Strep-F (5′-TACATTTCTTACATCTATGCGGACgacccctccaaggactcgaa-3′) and the C0 reverse primer. The primer NCM-oe-Strep-F contained an overlap of the regulatory sequences needed for CFPS, including the CrPV IRES site and the Mel signal peptide, in order to have a similar construct to LTB and LTB-Strep. Hence, a second PCR was performed in which the first PCR template was fused to the NCM-F primer.

The templates from the first PCR step and the NCM-F primer were applied in an equal molar template ratio of 12.5 nM in a standard HiFi PCR protocol.

### 4.5. DNA Template for Orthogonal tRNA 

The template DNA for the orthogonal tRNA, which was used for the subsequent transcription, was generated using a Taq PCR reaction (0.01 ng/µL plasmid DNA, dNTPs 0.2 mM each (Qiagen), MgCl_2_ 2.5 mM (Thermo Fisher Scientific, Rockford, IL, USA) Taq-polymerase 0.025 U/µL (Thermo Fisher Scientific), 1× Taq-buffer (Thermo Fisher Scientific)). A specific O-methyl primer pair (0.5 µM each) was used as previously described [44]. Finally, the PCR product was purified using Qiaquick PCR-Purification Kit (Qiagen) and analyzed by agarose gel electrophoresis. 

### 4.6. Generation of Orthogonal Components

The orthogonal aminoacyl-tRNA synthetase [44] (eAzPheRS), which is specific for p-propargyloxyphenylalanine (pPa) and p-azido-L-phenylalanine (AzF), was synthesized in the “RTS500 ProteoMaster *E. coli* HY Kit” (Biotechrabbit GmbH, Berlin, Germany). Synthesis, purification and storage of eAzPheRS were handled as previously described [17,44]. 

Specific transcripts of suppressor tRNAs were transcribed in vitro overnight in a batch-formatted reaction at 37  °C, using a PCR product as a DNA template (f.c. 8 µg/mL) as described in Zemella et al., 2009 [44]. The tRNA was purified using phenol-chloroform extraction with TRIzol-reagent (Life Technologies, Carlsbad, CA, USA). Subsequently, the purified tRNA was resuspended in ultrapure water and stored at −80 °C.

### 4.7. Cell-Free Protein Synthesis

Cell-free protein synthesis reactions were performed using translationally active lysates derived from cultured Chinese hamster ovary cells (CHO, DSMZ, Braunschweig, Germany) and cultured *Spodoptera frugiperda* 21 cells (*Sf*21, ECACC by Sigma Aldrich, Taufkirschen, Germany). Lysate preparation was performed as described previously [45,46,47]. For individual subunit expression, the plasmid was added at a final concentration (f.c.) of 60 ng/µL. For co-expression syntheses, the templates of the subunits were added in a 1:1 (A1:A2), 1:5 (A:B) or 1:1:5 (A1:A2:B) molar DNA ratio. Whenever a template that was derived from a PCR was used, the PCR template concentration was estimated according to the DNA quantification standard (100 ng/µL, 1000 bp; Gensura, San Diego, CA, USA). Furthermore, a no-template control (NTC), consisting of a translation mixture without any DNA template was used as a background control.

### 4.8. Batch-Based Reactions

Cell-free protein synthesis was performed in a coupled transcription/translation mode at final volumes ranging from 20 to 85 µL. Reactions were conducted according to previously described protocols [47,48]. Briefly, the DNA template was added to a reaction mixture composed of 40% (*v*/*v*) lysate, 100 µM amino acids and energy components. PolyG primer (f.c. 10 µM, IBA, Göttingen, Germany) was additionally supplemented. For further analyses, such as autoradiography and liquid scintillation counting, cell-free protein synthesis reactions were supplemented with radioactive ^14^C-leucine (f.c. 50 μM, specific radioactivity 66.67 dpm/pmol, Perkin Elmer, Baesweiler, Germany). The reactions were incubated for 3 h at 30 °C for CHO lysate or 27 °C for *Sf*21 lysate at an agitation of 500 rpm.

### 4.9. Repetitive Synthesis

The visualization of the co-translational translocation of the LTB-eYFP construct was undertaken after the repetitive synthesis scheme of the respective construct. Each cycle was performed in a 20 µL standard batch-based synthesis reaction in *Sf*21 lysate. Each synthesis took place for 3 h at 27 °C and 500 rpm. A total of three synthesis rounds were performed. After the first round, the microsomal vesicles that contained the protein of interest were separated from the soluble synthesis components by centrifugation (10 min, 16,000× *g*, 4 °C). This microsomal pellet was dissolved in a fresh reaction mixture as used for batch-based syntheses. The only exception was that the *Sf*21 lysate was depleted from microsomes by centrifugation. After the second synthesis, the microsomes were collected again and dissolved in a fresh reaction mixture without additional microsomes.

### 4.10. Orthogonal Translation

For site-specific incorporation of pPa and AzF, the respective ncAA (f.c. 2 mM, both from Iris Biotech GmbH, Marktredwitz, Germany), the pre-synthesized tRNA (f.c. 2.5 and 5 µM for *Sf*21 and CHO, respectively) were added to the standard coupled batch reaction as described above. A modified CHO lysate that integrated the eAzPheRS through stable transfection was used. Using this lysate, no eAzPheRS had to be separately supplemented.

### 4.11. Protein Fractionation

As the cholera toxin subunits harbor a Melittin signal sequence, a translocation of the synthesized protein into the microsomal vesicles that are present in the CHO and *Sf*21 lysate [42] was expected. Therefore, microsomal vesicles were harvested. After the synthesis reaction, the crude translation mixture (TM) was centrifuged (16,000× *g*, 10 min, 4 °C) resulting in the supernatant (SN1) containing the soluble subunits that were not translocated into the vesicles and the pelleted microsomes. The pellet was resuspended in phosphate-buffered saline (PBS, Merck, Darmstadt, Germany) containing 0.5% of the mild detergent Chaps (3-((3-cholamidopropyl) dimethylammonio)-1-propanesulfonate, Amresco, Solon, OH, USA), which was termed the microsomal fraction (MF). MF was incubated at room temperature under rigorous agitation for 45 min. Finally, the fraction was centrifuged (16,000× *g*, 10 min, 4 °C) resulting in the supernatant (SN2) containing the soluble subunits.

### 4.12. Fluorescent Labeling

Chemoselective labeling of pPa and AzF was performed. Shortly, a copper-catalyzed azide-alkyne cycloaddition was performed for constructs that incorporated pPa by adding 3[tris(3-hydroxypropyltriazolylmethyl)amine (ThPTA, f.c. 600 µM, Sigma Aldrich), sodium ascorbate (f.c. 5 mM, Sigma Aldrich), copper sulfate (f.c. 50 µM, Sigma Aldrich) and sulfo-Cy 5-azide (f.c. 5 µM, Lumiprobe, Hannover, Germany). The reaction took place in the dark at 25 °C for 90 min. A Staudinger ligation was used to label constructs that incorporated AzF. The sample was mixed with Dylight 632-Phosphine (f.c. 5 µM, Dyomics GmbH, Jena, Germany) and incubated in the dark for 90 min at 25 °C.

LTB-strep and Strep were labeled using a fluorescent dye that was fused to a biotin relying on the strep-biotin binding. Hence, the fluorescent dye Biotin-Atto488 (f.c. 1 µM, Sigma Aldrich) was mixed with the sample and incubated in the dark for 90 min at 25 °C.

### 4.13. Quantitative Protein Analysis

Total protein yield of synthesized proteins was determined by using hot trichloroacetic acid (TCA, Carl Roth GmbH, Karlsruhe, Germany) precipitation and liquid scintillation quantification as previously described [20]. The total protein yield for co-expressed subunits was estimated using the sum of the molecular weight and the sum of the number of leucines of all expressed subunits according to the previously published protocol [20].

### 4.14. Qualitative Protein Analysis

Proteins were either precipitated in acetone as described previously [20] or the sample was directly mixed with LDS sample buffer (NUPAGE LDS sample buffer (Invitrogen, Thermo Fisher Scientific). If not stated otherwise, samples were heated to 70 °C for 10 min. Sodium dodecyl sulfate polyacrylamide gel electrophoresis (SDS-PAGE) using precast gels (NuPAGE, 10% Bis-Tris, Life technologies) and autoradiography were performed according to previous protocols [12,17,20]. 

In-gel fluorescence was performed for fluorescently labeled constructs directly after SDS-PAGE. The fluorescence scan was performed using the Amersham RGB Typhoon (GE Healthcare, Little Chalfont, Buckinghamshire, UK, DyLight632-Phosphine und Cy5-dyes: excitation 633 nm, emission 670 nm, Biotin-Atto488 dye, excitation 488 nm, emission 525 nm)

The SeeBlue PreStained Protein standard and SeeBlue Plus 2 PreStained Protein standard (Thermo Fisher Scientific) were used.

### 4.15. LTB-eYFP Fluorescence Analysis

An amount of 5 µL of the pre-synthesized protein was diluted in 20 µL PBS and added onto an µ-IBIDI-slide (Ibidi, Planegg, Germany). The translocation was assessed by confocal laser scanning microscopy (CLSM). The laser scanning microscope unit (LSM 510, Carl Zeiss Microscopy GmbH, Oberkochen, Germany) was used, and eYFP was exited with an argon laser at 488 nm. After passing a long-pass filter with a wavelength of 505 nm, the emitted light was captured using a photomultiplier.

### 4.16. Cell-Based Activity Assessment

For functional analysis, the effect of the AB_5_ complex and individual subunits was investigated in CHO-K1 cells (DSMZ, Braunschweig, Germany). In an initial experiment, the cell suspension (CHO-K1 cells in DMEM (Sigma Aldrich), 1% fetal bovine serum (Merck) and 1% Penicillium/Streptomycin (Merck)) with a concentration of 4000 cells/well were added to each well of a 96-well plate (Sarstedt, Nümbrecht, Germany). In the following experiments, 24-well plates (Sarstedt) with 25,000 cells/well were used. Cells were seeded shortly before adding the toxin and incubated at 37 °C and 5% CO_2_-flow. The toxins were synthesized in a batch-based reaction with either CHO or *Sf*21 lysate. All toxins were synthesized in two reactions, one reaction with additional ^14^C-labeled leucine, and the other reaction without ^14^C-leucine. The SN2 fraction of the labeled protein was quantitatively and qualitatively analyzed as described above. Toxin concentrations of the radiolabeled toxins were calculated and, consequently, assumed for non-labeled toxins, as both reactions were prepared simultaneously. In order to allow for the same detergent concentration of the final sample that was added to the cells, all samples were diluted and filled up to the same volume of 0.5% CHAPS/PBS and medium. The mixture of the toxin, CHAPS/PBS and medium was added to each well. A no-template control (NTC) was added by using a volume equivalent to the toxin fragment. Morphological changes were documented using a light microscope (Leica, Wetzlar, Germany) and phase contrast micrographs were captured with a CCD camera (Leica) after 48 h. 

The cytotoxicity of the toxins was assessed by the CellTox Green Cytotoxicity Assay (Promega, Walldorft, Germany) according to the manufacturer’s protocol in a 96-well plate (Sarstedt). Cells were cultivated as described for morphological assays. CHO-K1 cells were incubated with toxins for 48 h. 

A univariate analysis of variance (ANOVA) was used to assess the significant changes in the cytotoxicity assay. Significance was tested according to Bonferroni. Statistically significant changes were indicated in the Figures with *.

## Data Availability

All relevant data are within the paper and its Supporting Information files.

## Supplementary Materials

The following supporting information can be downloaded at: https://www.mdpi.com/article/10.3390/toxins14040233/s1, Figure S1. Complex formation of Ctx and LT in *Sf*21. Figure S2. CHO-K1 cells 24 h after LT addition. Figure S3. CHO-K1 cells after LT addition. Figure S4. CHO-K1 cells treated with LTAB-Strep control subunits. Figure S5. CHO-K1 cells after LT addition for cytotoxicity assessment. Figure S6. Single Ctx subunits supplemented to CHO-K1 cells. Figure S7. CHO-K1 cells treated with CtxAB derived from *Sf*21. Figure S8. CHO-K1 cells after Ctx addition for cytotoxicity assessment. Figure S9. CtxAamb subunits labeled with sulfo-Cy5 azide.
