# Supplementary material for: Cell-Free Systems Enable the Production of AB5 Toxins for Diagnostic Applications
Source: Toxins (Basel). 2022 Mar 23;14(4):233. doi: 10.3390/toxins14040233 (PMC9027097; doi:10.3390/toxins14040233)
Supplement: Supplementary file 1 [file toxins-14-00233-s001.zip › toxins-1599977-supplementary.pdf]

Supplementary Materials: Cell-Free Systems Enable the Production of AB5 Toxins for Diagnostic Applications

Franziska Ramm, Lena Jack, Danny Kaser, Jeffrey L. Schloßhauer , Anne Zemella and Stefan Kubick

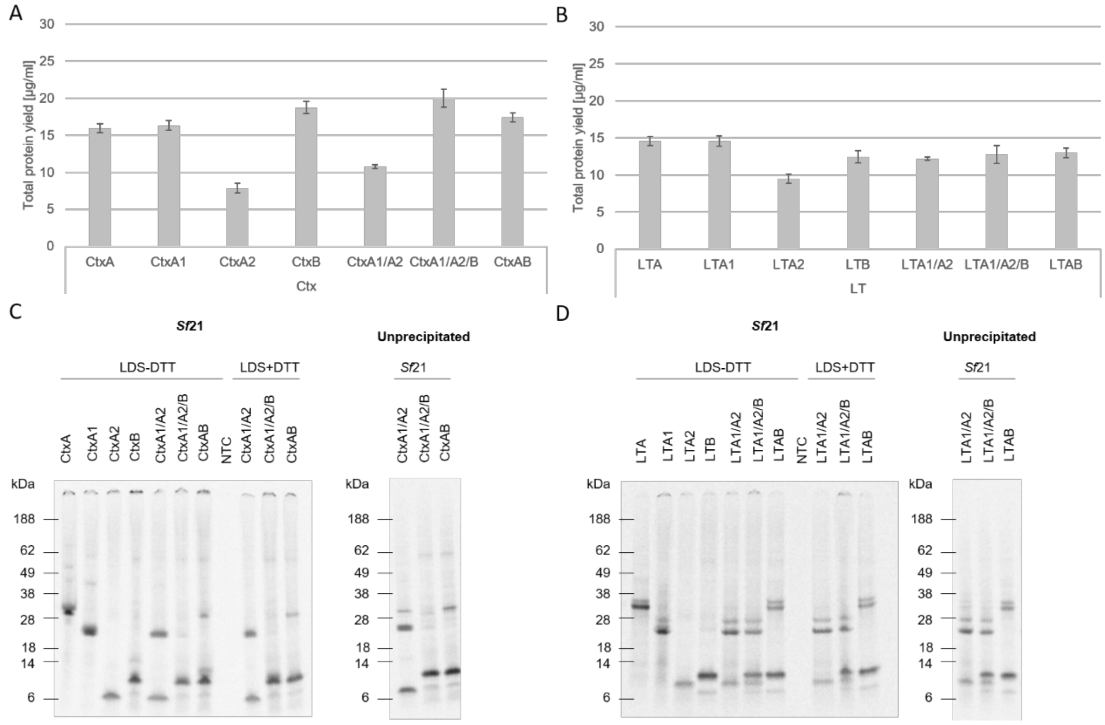

Figure S1. Complex formation of Ctx and LT in *Sf*21. Single subunits and co-expressed subunits of Ctx (A) and LT (B) were synthesized in a *Sf*21 lysate. Total protein yields of *de novo* synthesized protein were analyzed by liquid scintillation for the translation mixture (TM). Standard deviations were calculated from triplicate analysis. Autoradiograph of Ctx (C) and LT (D) subunits after the single subunit synthesis and co-expression of subunits.

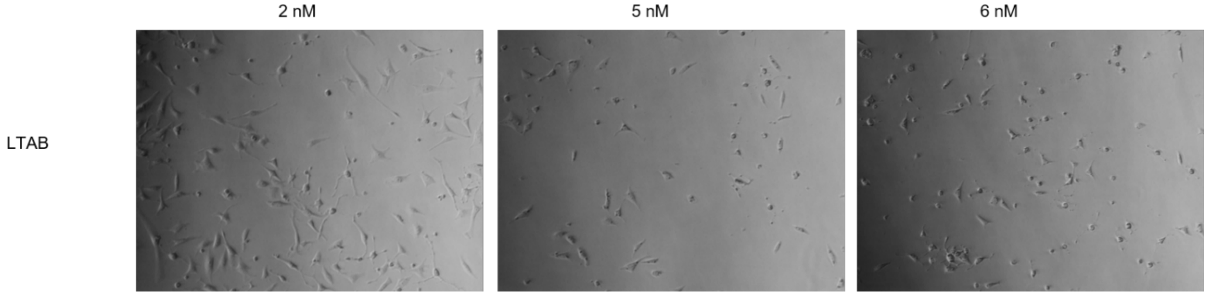

Figure S2. CHO-K1 cells 24 h after LT addition. Co-expressed LTAB was synthesized in *Sf*21 lysate and supplemented to CHO-K1 cells (4,000 cells/well) in a 96-well plate. Phase contrast photographs were taken after 24 h.

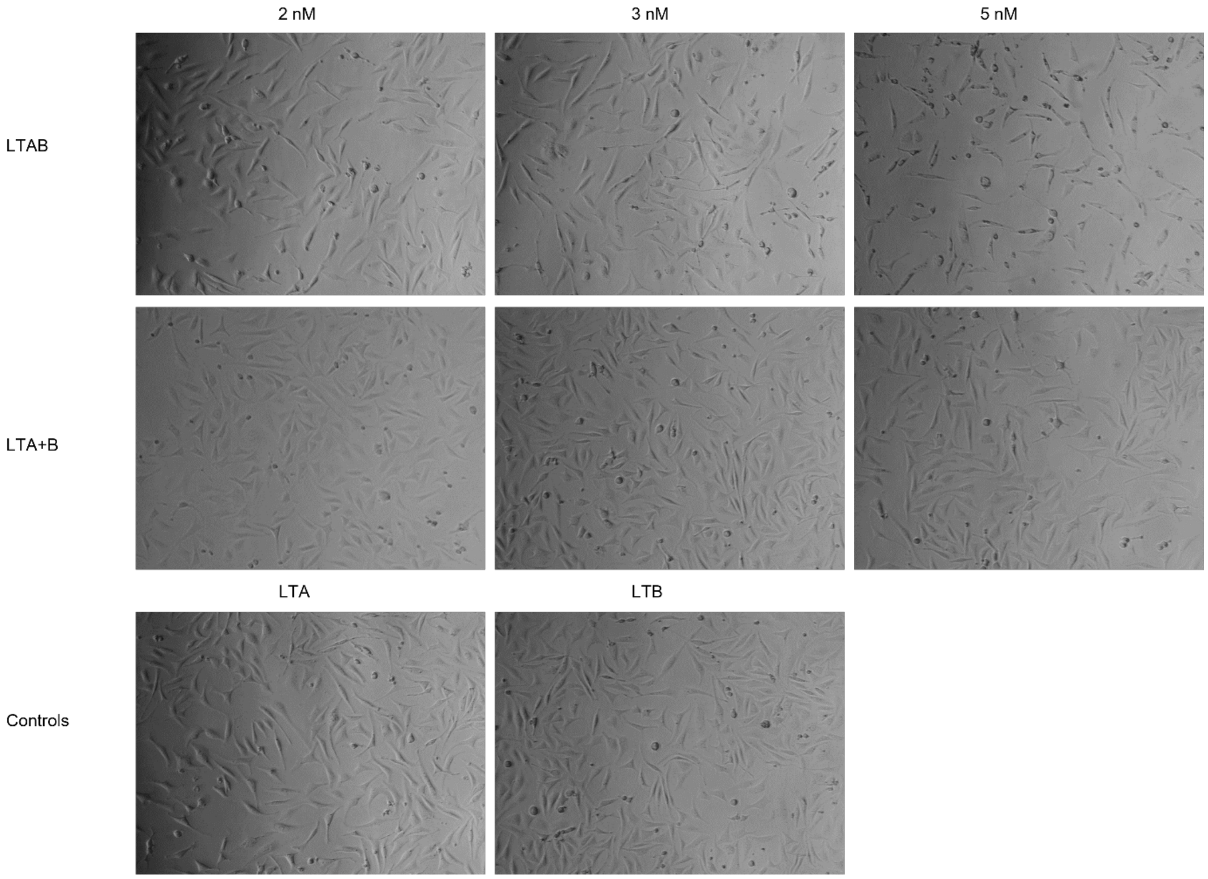

Figure S3. CHO-K1 cells after LT addition. Single subunits and co-expressed LTAB and pre-synthesized LTA and B which were mixed afterwards to form the multimer (LTA+B) were synthesized in *Sf*21 lysate and supplemented to CHO-K1 cells (4,000 cells/well) in a 96-well plate. Phase contrast photographs were taken after 48 h of incubation. LTA and B were supplemented at 6 nM.

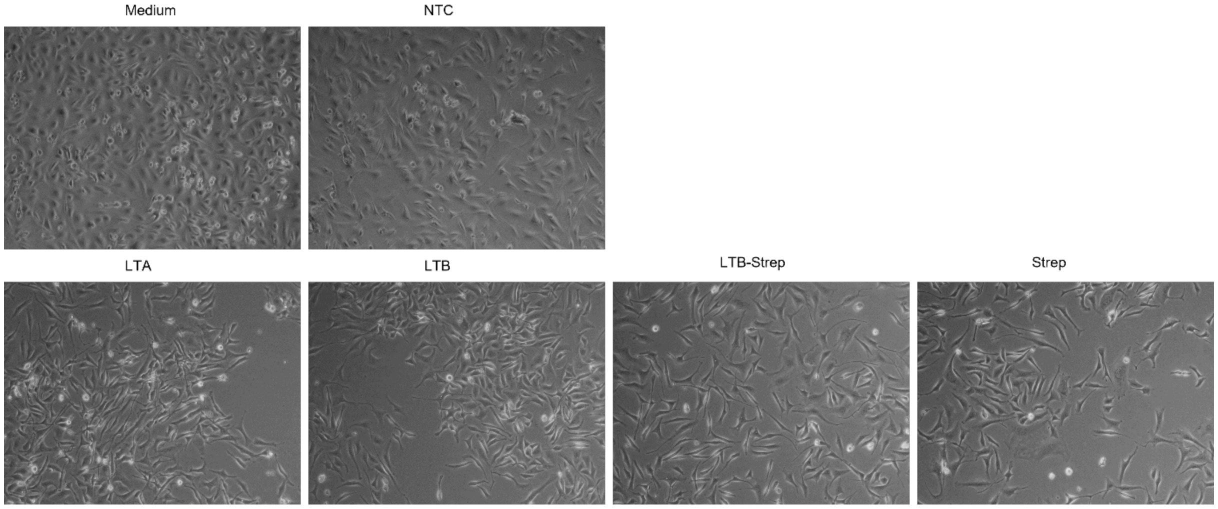

Figure S4. CHO-K1 cells treated with LTAB-Strep control subunits. Single subunits were synthesized in *Sf*21 lysate and supplemented to CHO-K1 cells (25,000 cells/well) in a 24-well plate. Phase contrast

photographs were taken after 48 h of incubation. Subunits and the volume equivalent NTC were supplemented at a concentration of 5 nM.

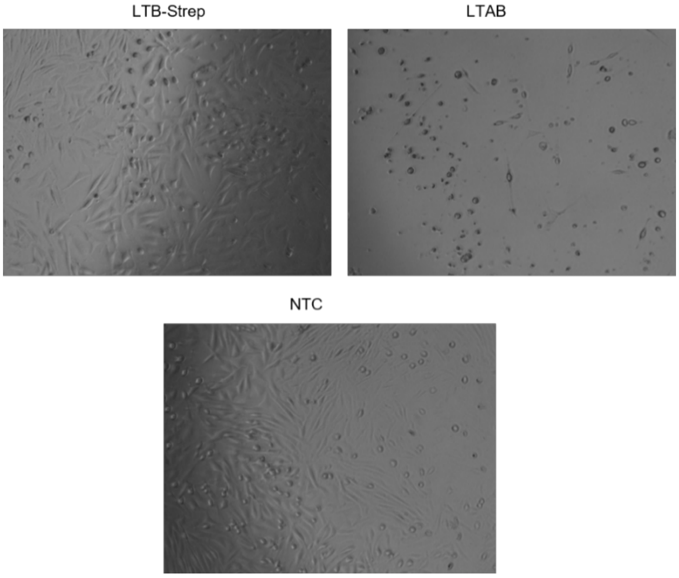

Figure S5. CHO-K1 cells after LT addition for cytotoxicity assessment. LTB-Strep, co-expressed LTAB and NTC were synthesized in *Sf*21 lysate and supplemented to CHO-K1 cells in a 96-well plate for cytotoxicity assessment. Phase contrast photographs were taken after 48 h of incubation.

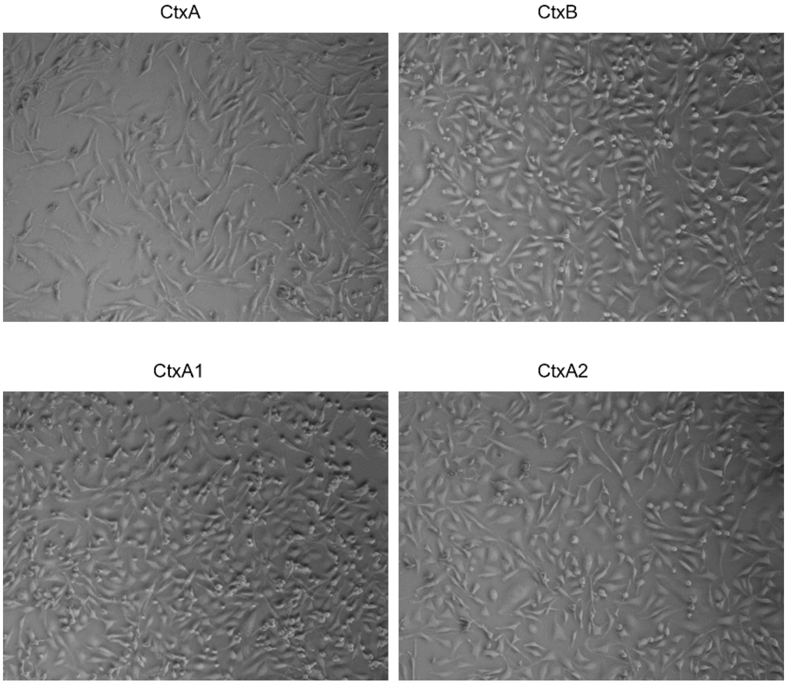

Figure S6. Single Ctx subunits supplemented to CHO-K1 cells. Ctx subunits were synthesized in CHO lysate and were supplemented to CHO-K1 cells (25,000 cells/well) in a 24-well plate. Phase contrast photographs were taken after 48 h of incubation.

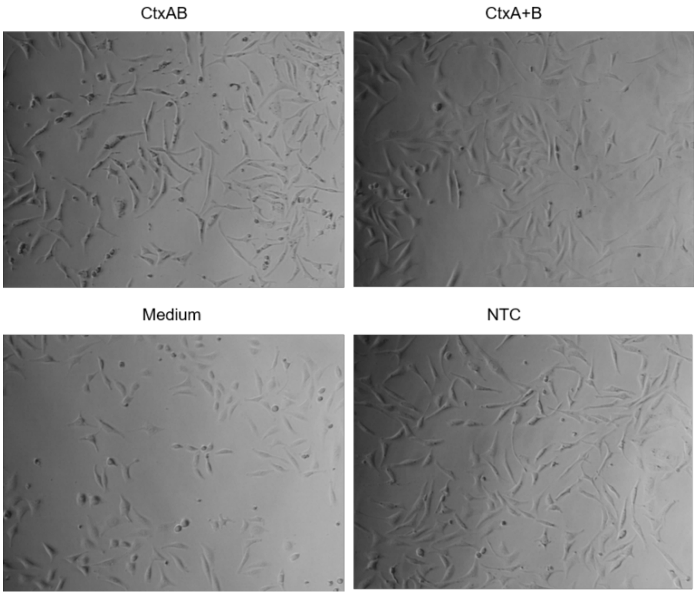

Figure S7. CHO-K1 cells treated with CtxAB derived from *Sf*21. Co-expressed CtxAB and pre-synthesized CtxA and B which were mixed afterwards to form the multimer (CtxA+B) were synthesized in *Sf*21 lysate and supplemented to CHO-K1 cells (4,000 cells/well) in a 96-well plate. Phase contrast photographs were taken after 48 h of incubation.

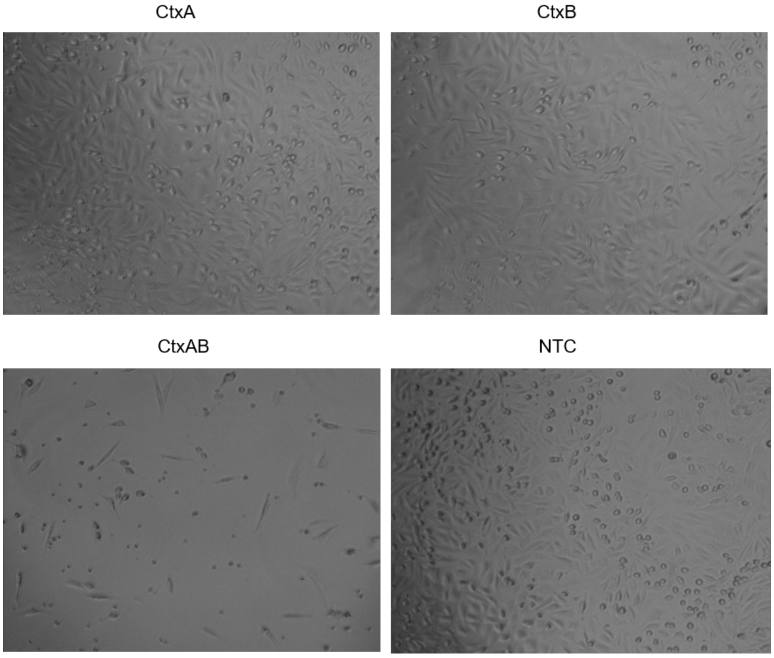

Figure S8. CHO-K1 cells after Ctx addition for cytotoxicity assessment. CtxA, CtxB, co-expressed CtxAB and NTC were synthesized in CHO lysate and supplemented to CHO-K1 cells in a 96-well plate for cytotoxicity assessment. Phase contrast photographs were taken after 48 h of incubation.

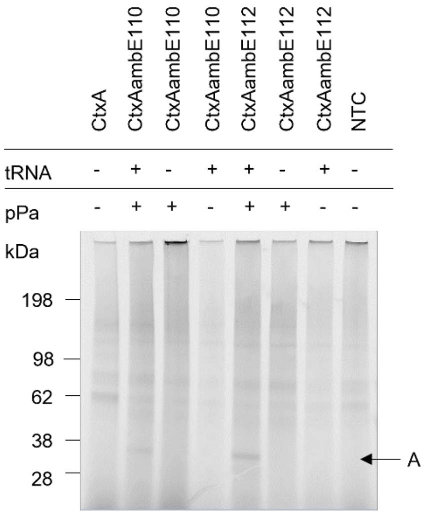

Figure S9. CtxAamb subunits labeled with sulfo-Cy5 azide. In-gel fluorescence of CtxAamb mutants labeled with sulfo-Cy5 azide after orthogonal translation in an optimized CHO system.
